# Supplementary material for: Integrated Analysis of the Lung Microbiome and Metabolome Reveals Associations Between Amino Acid Metabolism and Pulmonary Fibrosis in a Bleomycin-Induced Mouse Model
Source: Int J Mol Sci. 2026 Jun 30;27(13):5895. doi: 10.3390/ijms27135895 (PMC13362081; doi:10.3390/ijms27135895)
Supplement: Supplementary file 1 [file ijms-27-05895-s001.zip › result/4.MetDiffAnalysis/B_28d.vs.C_28d/B_28d.vs.C_28d_all_zscore.pdf]

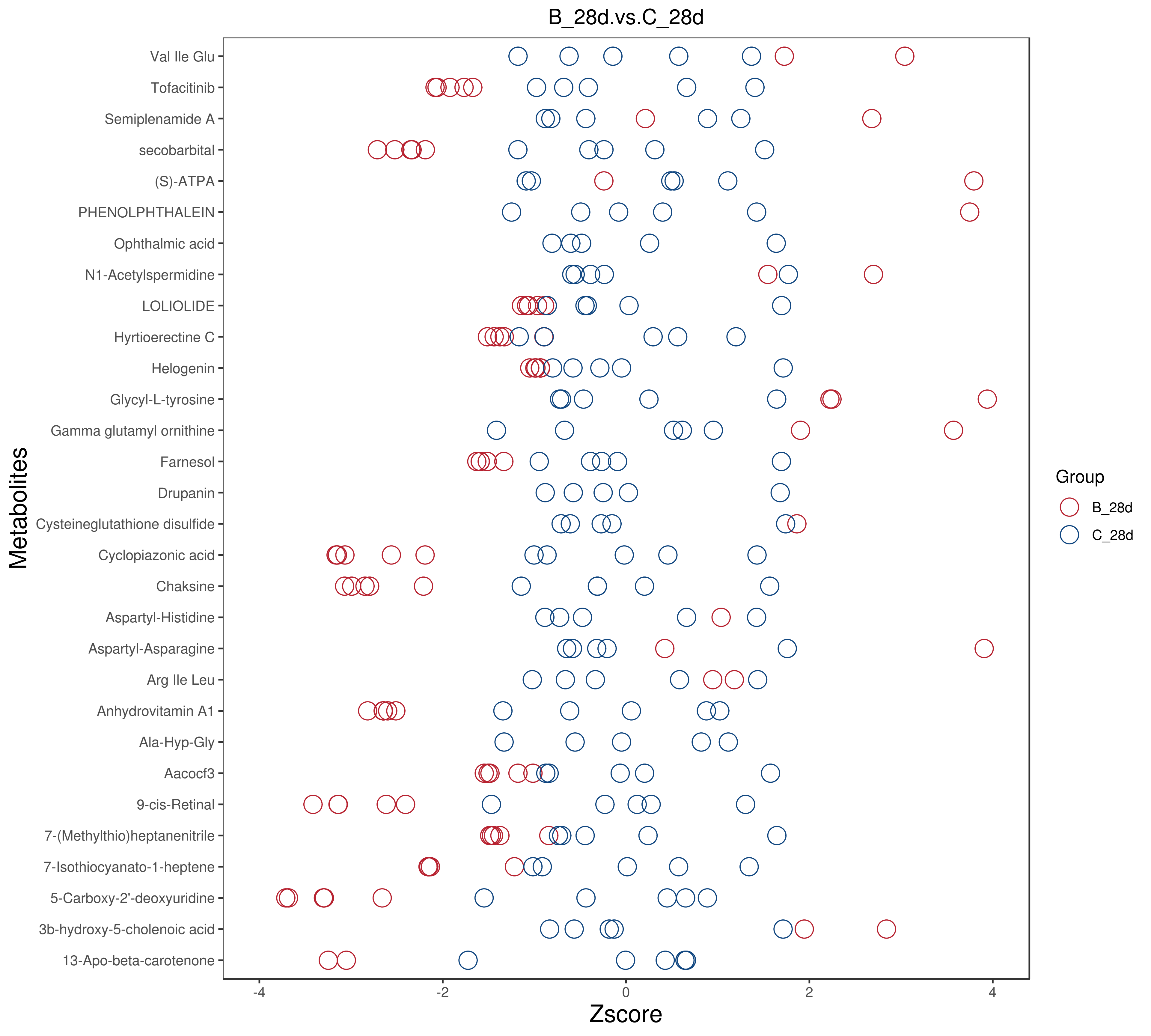
B_28d.vs.C_28d
Val Ile Glu
Tofacitinib
Semiplenamide A
secobarbital
(S)-ATPA
PHENOLPHTHALEIN
Ophthalmic acid
N1-Acetylspermidine
LOLIOLIDE
Hyrtioerectine C
Helogenin
Glycyl-L-tyrosine
Gamma glutamyl ornithine
Farnesol
Drupanin
Cysteineglutathione disulfide
Cyclopiazonic acid
Chaksine
Aspartyl-Histidine
Aspartyl-Asparagine
Arg Ile Leu
Anhydrovitamin A1
Ala-Hyp-Gly
Aacocf3
9-cis-Retinal
7-(Methylthio)heptanenitrile
7-Isothiocyanato-1-heptene
5-Carboxy-2'-deoxyuridine
3b-hydroxy-5-cholenoic acid
13-Apo-beta-carotenone
Metabolites
-4
-2
0
2
4
Zscore
Group
B_28d
C_28d
